# MicroRNA - a contributor to age-associated neural stem cell dysfunction?

**DOI:** 10.18632/aging.100297

**Published:** 2011-03-12

**Authors:** Muhammad Amir Khan, Dieter Chichung Lie

**Affiliations:** Research Group Adult Neural Stem Cells and Neurogenesis, Institute of Developmental Genetics, Helmholtz Zentrum München, German Research Center for Environmental Health, 85764 Munich-Neuherberg, Germany

In the adult mammalian brain, new neurons are continuously generated from neural stem cells in the subventricular zone (SVZ) of the lateral ventricles and the subgranular zone (SGZ) of the hippocampus [1]. Increasing evidence for the functional importance of adult-generated neurons in these regions for learning, complex behavior, and mood regulation, as well as a potential link of impaired adult neurogenesis to cognitive deficits in ageing and neurodegeneration have sparked great interest in the regulatory mechanisms underlying the coordinated generation of new functional neurons. Neurogenesis includes self-renewal and fate specification of neural stem cells, migration and maturation of young neurons, and functional integration of new neurons into the neural circuitry [1]. A large body of work has demonstrated that neurogenesis is regulated in a complex manner by the dynamic interplay of cell-extrinsic signals derived from the neurogenic “niche” and cell-intrinsic transcriptional and epigenetic regulators. More recently, microRNAs (miRNAs) have emerged as potent modulators of adult neurogenesis. miRNA 132 has been linked to the maturation and functional integration of newly generated hippocampal neurons and – in this context – is potentially regulated by hippocampal network activity and CREB-signalling [2]. Several miRNAs have also been implied in controlling the balance of stem cell maintenance and differentiation - a crucial checkpoint for sustaining adult neurogenesis throughout adulthood. In this context, miRNA-137 [3] and miRNA-184 [4] have been found to promote stem cell maintenance at the expense of differentiation, while miRNA-9 [5], miRNA lethal-7b (let-7b) [6, 7], and miRNA-124 [8] tip the balance from stem cell maintenance towards differentiation through the negative regulation of cyclin D1 and the stem cell maintenance factors TLX, Hmga2, and Sox9. Intriguingly, the latter factors are transcriptional regulators and thus are likely to control the expression of larger sets of genes, which may explain the profound effects of those miRNAs in stem cell maintenance and differentiation.

In the February issue of Aging Brett and colleagues link the microRNA cluster miR-106b~25 to neural stem cell expansion and neuronal differentiation [9]. In this work, they demonstrate that miR-106b~25 promotes proliferation in primary neural stem cell cultures. Moreover they report that overexpression of miR-106b~25 enhances neural stem cell differentiation toward the neuronal lineage. Although the in vitro modulatory effects of miRNA-106b~25 on neural stem cell behaviour are relatively modest, the authors made several notable observations that imply this miRNA cluster as a critical regulator of adult neurogenesis and warrant further investigation of its function and regulation: Firstly, *in silico* predictions suggest that miRNA-25 may be involved in the modulation of transforming growth factor β (TGFβ)/bone morphogenic protein (BMP) and insulin/IGF signalling, i.e., signalling pathways that control neural stem cell quiescence, proliferation, and fate as well as age-related stem cell dysfunction in other organs. Secondly, the authors identify a functional FoxO3 binding site near the promoter for miR-106b~25, which modulates the activity of the miRNA cluster. Recent work identified FoxO transcription factors as crucial regulators of stem cell maintenance in the hematopoietic and the adult central nervous system, whose loss results in premature depletion of the stem cell pool. Notably, FoxO transcription factor-dependent pathways control ageing and longevity in *C.elegans* and *Drosophila melanogaster* and certain FoxO3 gene variants are associated with increased lifespan in people [10].

Hence, the miRNA-106b~25 cluster may emerge as an important modulator of ageing in neural stem cells and the neurogenic niches. The *in vivo* function of miRNA-106b~25 in neural stem cell behaviour and neurogenesis has to be determined and it will be particularly interesting to determine the impact of ageing on the activity of the miRNA-106b~25 cluster in neural stem cells and their progeny. Moreover, it will be important to understand if and how the miRNA-106b~25 cluster is controlled by signals derived from the neurogenic environment, given the evidence that major shifts in pathway activities contribute to stem cell dysfunction during ageing. In the long run, determining functional targets of miRNA-106b~25 in the adult neurogenic lineage may reveal novel pathways in the control of neurogenesis, which may be harnessed for treatment of age-associated cognitive deficits.
